# Trichothecene Mycotoxins Inhibit Mitochondrial Translation—Implication for the Mechanism of Toxicity

**DOI:** 10.3390/toxins3121484

**Published:** 2011-12-02

**Authors:** Mohamed Anwar Bin-Umer, John E. McLaughlin, Debaleena Basu, Susan McCormick, Nilgun E. Tumer

**Affiliations:** 1 Department of Plant Biology and Pathology, School of Environmental and Biological Sciences, Rutgers University, New Brunswick, NJ 08901, USA; Email: binumer@rutgers.edu (M.A.B.-U.); mclaughj@rci.rutgers.edu (J.E.M.); debabasu@eden.rutgers.edu (D.B.); 2 Bacterial Foodborne Pathogens and Mycology Unit, National Center for Agricultural Utilization Research, United States Department of Agriculture, Agricultural Research Service, Peoria, IL 61604, USA; Email: susan.mccormick@ars.usda.gov

**Keywords:** trichothecenes, deoxynivalenol, T-2 toxin, diacetoxyscirpenol, Fusarium, mitochondria, ribosomes, translation, fusion, Fission, ROS

## Abstract

Fusarium head blight (FHB) reduces crop yield and results in contamination of grains with trichothecene mycotoxins. We previously showed that mitochondria play a critical role in the toxicity of a type B trichothecene. Here, we investigated the direct effects of type A and type B trichothecenes on mitochondrial translation and membrane integrity in *Saccharomyces cerevisiae*. Sensitivity to trichothecenes increased when functional mitochondria were required for growth, and trichothecenes inhibited mitochondrial translation at concentrations, which did not inhibit total translation. *In organello* translation in isolated mitochondria was inhibited by type A and B trichothecenes, demonstrating that these toxins have a direct effect on mitochondrial translation. In intact yeast cells trichothecenes showed dose-dependent inhibition of mitochondrial membrane potential and reactive oxygen species, but only at doses higher than those affecting mitochondrial translation. These results demonstrate that inhibition of mitochondrial translation is a primary target of trichothecenes and is not secondary to the disruption of mitochondrial membranes.

## 1. Introduction

Trichothecenes are foodborne toxins produced by various fungi including the plant pathogen *Fusarium graminearum*, which causes head blight or scab of wheat and barley, resulting in yield reduction and contamination of grains with trichothecene mycotoxins [1]. Fusarium head blight (FHB) has re-emerged worldwide as a disease of economic importance due to changes in climate and agricultural practices [2]. The scab epidemic of 1990s resulted in wheat and barley losses close to $3 billion in the US alone [3]. 

Trichothecenes are structurally related mycotoxins characterized by two functionally critical features: a double bond at C9 and C10 and an epoxide ring at C12 and C13 [1]. Based on their substitution pattern of specific functional groups, trichothecenes are divided into four groups with varied toxicity that is related to their structure [1,4,5]. Type A trichothecenes T-2 toxin (T-2) and diacetoxyscirpenol (DAS) have a hydroxyl group, an ester group or no side chain at C8 while type B trichothecenes (DON, trichodermin, trichothecin) have a keto group instead. Type C trichothecenes (crotocin) have a second epoxide ring, while type D trichothecenes (verrucarin, satratoxin) contain a macrocyclic ring [1,4]. Of the 200+ trichothecenes identified, DON and T-2 are toxicologically the most relevant and widely studied. 

Trichothecenes were first identified as inhibitors of translation that target the peptidyl transferase center [6]. However, they are known to have multiple effects on eukaryotes, including inhibition of DNA, RNA synthesis, cell division, membrane structure and integrity and mitochondrial function [7]. It is not clear if these are primary or secondary effects of inhibition of cytosolic translation. Differences in the mechanism of translation inhibition between the various types of trichothecenes have been reported [8]. T-2 is reported to inhibit translation by preventing formation of the initial peptide bond, while trichodermin, DON and trichothecin (Tcin) inhibit the elongation step [9]. Trichothecenes were shown to activate a cellular stress response, termed the ribotoxic stress response [10], which is also activated by ribosome inactivating proteins (RIPs) that damage the large rRNA [11]. Trichothecenes were recently shown to promote cleavage of 28S rRNA in mammalian cells, a hallmark feature of agents that cause ribotoxic stress [12]. The amphipathic nature of trichothecenes allows these toxins to cross the cell membrane and interact with different organelles such as the mitochondria [13,14], endoplasmic reticulum (ER) [15] and chloroplast [16]. Hence the toxicity of trichothecenes may not only be the result of a translation arrest, but likely involves other cellular processes. 

Previous studies have suggested a role for mitochondria in trichothecene toxicity [13,14,17]. Transcription profiles of genes associated with mitochondria were altered in *Saccharomyces cerevisiae* treated with T-2 [18,19], and mitochondria-dependent apoptotic pathways were activated in mammalian cells exposed to T-2 [17]. We established *Saccharomyces cerevisiae* as a model to study the mechanism of toxicity of trichothecenes and in a genome-wide screen, we identified mitochondria associated genes as the largest group of deletions that conferred resistance to 4 µM trichothecin (Tcin) [20]. We showed that mitochondrial translation was inhibited to a greater extent in the wild type strain than in the deletion mutants that showed resistance to Tcin [20]. Furthermore, treatment of yeast cells with Tcin led to the fragmentation of the tubular mitochondrial network, supporting a role for Tcin in disruption of mitochondrial membrane morphology [20]. To date, the mechanistic basis of trichothecene toxicity is not fully understood. Furthermore it is not clear if the effects on mitochondria are primary or secondary to the inhibition of cytosolic translation. In this study, to determine if trichothecenes have a direct effect on mitochondrial translation, we examined translation in isolated mitochondria and in intact cells in the presence of different trichothecenes. Our results demonstrate for the first time that type A and type B trichothecenes inhibit translation in isolated mitochondria. We further show that trichothecenes have time and dose-dependent effects on mitochondrial membrane potential and generation of reactive oxygen species (ROS), but only at doses higher than those inhibiting mitochondrial translation. These results demonstrate that organellar translation is a primary target of both type A and type B trichothecenes and implicate inhibition of mitochondrial translation in trichothecene toxicity. 

## 2. Materials and Methods

### 2.1. Yeast Strains

Yeast strain BY4743 (*MATa/α*, *his3Δ1/his3Δ1*, *leu2Δ0/leu2Δ0*, *LYS2/lys2Δ0*, *met15Δ0/MET15*, *ura3Δ0/ura3Δ0*) was used for all experiments except mitochondria were isolated from W303 (*MATa/α*, *leu2-3*, *112 trp1-1*, *can1-100*, *ura3-1*, *ade2-1*, *his3-11*, *15*).

### 2.2. Trichothecene Isolation

Tcin was isolated from *Trichothecium roseum* and prepared as described previously [20]. 4,15-Diacetoxyscirpenol was isolated from YEPD cultures of *F. sporotrichioides* strain 1716 cos9-1 #1 (a mutant of *F. sporotrichioides* NRRL3299) [21], and purified on a silica gel column eluted with 5% methanol in dichloromethane. T-2 toxin was isolated from YEPD cultures of *F. sporotrichioides* strain 5493 cos9-1 #11 (a mutant of *F. sporotrichioides* NRRL3299) [21], and purified on silica gel columns eluted with 5% methanol in dichloromethane and hexane/ethyl acetate/methanol (12:12:1).

### 2.3. Growth Assay

Wild type BY4743 cells were grown for 18 h in yeast peptone (YP) media (Fischer Scientific, Fairlawn, NJ) supplemented with either 2% dextrose (hereon referred to as YPD) or 3% glycerol (hereon referred to as YPG) at 200 rpm at 30 °C. Trichothecenes were serially diluted (2X) in YPD or YPG media ranging from 0 to 200 µM and 0 to 2500 µM for T-2 and DAS respectively. Growth at OD_600_ was measured using the SpectraMax^®^ Plus384 (Molecular Devices, Sunnyvale, CA). Rho^0^ versions of BY4743 were generated and verified as described previously [20]. 

### 2.4. Analysis of Total Translation

Cells were grown in synthetic methionine dropout (SD-Met) media (Yeast Nitrogen Base w/o Amino Acids & Ammonium Sulfate, all amino acids except methionine) containing MSG as the nitrogen source and 2% raffinose, which prevents glucose repression and enhances respiration. Cultures grown to an OD_600_ of 0.2-0.3 were then split into two: one-half was treated with the specified amount of trichothecenes in ethanol and the other half was treated with an equivalent amount of ethanol. Treatments were carried out for the specified amount of time, shaking at 30 °C. At the end of the trichothecene treatment, OD_600_ was measured and 3 OD_600_ cells washed with minimal media by a quick spin (10,000 g for 1 min) and resuspended in 500 µL SD-Met (+2% raffinose). To each sample, 1 μL [35S]-Met (Perkin-Elmer, NEG-009A, >1000 Ci/mmol) was added. The reaction was stopped after 20 min by washing and resuspending cells in 500 µL (20 mM) cold methionine and 75 µL Rodel Mix (560 µL 5 M NaOH, 0.11 mL β-mercaptoethanol, 0.76 mL H_2_O, 0.075 mL 1 mM PMSF). An equal volume of 50% TCA (trichloroacetic acid) was added to the mix and filtered through 2.4 cm glass fiber filters (grade 691, VWR). Filters were then washed, once each, with 5% TCA and 95% ethanol and scintillation counts per minute (CPM) measured. CPM readings were finally normalized to OD_600_ to indicate total translation.

### 2.5. Analysis of Mitochondrial Translation

The mitochondrial translation assay was done as outlined in Section 2.4 with some modifications as described previously [22]. Prior to the addition of [35S]-Met, 20 µL (7.5 mg/mL) freshly prepared cycloheximide was added to each sample to selectively inhibit cytosolic translation and incubated for 5 min. Following cycloheximide treatment, [35S]-Met was added as described in Section 2.4 and incorporation was measured and normalized to OD_600_.

### 2.6. Mitochondria Isolation from Yeast

Mitochondria were isolated from yeast as described previously [23]. Briefly 10 g (wet weight) cells were collected from an overnight culture grown, in YP media supplemented with 2% lactate, to 1-2 OD_600_. All centrifugation steps, unless otherwise noted, were carried out for 5 min at 2500 g. The pellet was washed with H_2_O and incubated in freshly prepared TD buffer (100 mM Tris-SO_4_, pH 9.4, 10 mM DTT) for 5 min with gentle shaking. The pellet was then resuspended in SP buffer (1.2 M sorbitol, 20 mM potassium phosphate, pH 7.4) to which zymolyase was added for spheroplast formation. The spheroplasts were carefully collected and resuspended in 2X SHP buffer (1.2 M sorbitol, 40 mM HEPES-KOH, pH 7.4, 1 mM PMSF) to which equal volume of ice-cold H_2_O containing 1 mM PMSF was added. The resulting suspension was then carefully homogenized with a glass homogenizer. The homogenate was then centrifuged twice at 4 °C and the supernatant collected from each spin was combined and centrifuged for further 10 min at 12,000 g at 4 °C. The resulting pellet was resuspended in 1X SH buffer (0.6 M sorbitol, 20 mM HEPES-KOH, pH 7.4) for the *in organello* translation assay after protein quantification by the Bradford assay [24].

### 2.7. Mitochondrial *in Organello* Translation Assay

*In organello* translation, using isolated yeast mitochondria, was done as described previously [23] with some modifications. All incubations were done at 30 °C. Briefly, following a ten minute treatment with trichothecenes, 1 µL [35S]-Met was added to 20 µg of freshly isolated mitochondria, resuspended in the 1X SH buffer and incubated for 20 min. Labeling was stopped by the addition of 10 µL (200 mM) cold methionine and incubation for 5 min. Mitochondria were collected by centrifugation for 10 min at 20,000 g at 4 °C. The pellet was washed with 1X SH buffer and then filtered through 2.4 cm glass fiber filters. Filters were washed once each with 5% TCA and 95% ethanol and scintillation counts per minute (CPM) were measured. Readings were expressed as CPM/µg mitochondrial protein.

### 2.8. Staining, Microscopy &amp; Image Analysis

Mitochondrial morphology was examined using an epifluorescence microscope (Olympus BX41). BY4743 cells were transformed with pVT100U-mtGFP, which contains the green fluorescent protein (GFP) targeted to the mitochondria, with the presequence from the subunit 9 of the F0-ATPase of *Neurospora* *crassa*, as described previously [25]. Trichothecene-treated and untreated cells were stained with 2',7'-dichlorfluorescein-diacetate (DCFH*-*DA) for ROS generation and MitoTracker Red CMXRos for mitochondrial membrane potential according to manufacturer’s protocol. Stained cells were then examined with the Olympus BX41 microscope. All images were captured and analyzed using the MetaMorph^®^ Microscopy Automation & Image Analysis software (Molecular Devices, Sunnyvale, CA). 

### 2.9. Flow Cytometry

Trichothecene-treated and untreated cells, following staining with the appropriate dyes, were analyzed using the Accuri C6 Flow Cytometer^®^ (Accuri Cytometers Inc., Ann Arbor, MI). For each sample 25-50,000 events were recorded. Channel gating and histogram plots were made using the CFlow Plus Analysis software (Accuri Cytometers Inc., Ann Arbor, MI). Changes in MitoTracker Red and DCFH-DA fluorescence were detected using the FL1 and FL3 channel respectively.

### 2.10. Data Analysis &amp; Graphing

Data from the growth and translation assays were analyzed and the graphs were plotted using Microsoft Excel. 

## 3. Results

### 3.1. Mitochondria Are Important for Sensitivity to Type A Trichothecenes

**Figure 1:**
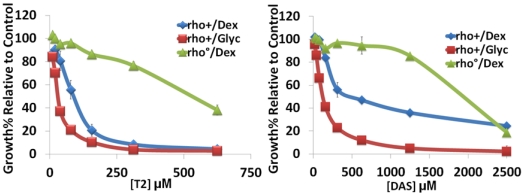
Growth of wild type BY4743 cells (rho^+^ & rho^0^) in media containing dextrose *vs.* glycerol. Rho^+^ and rho^0^ cells were grown in liquid media supplemented with 2% dextrose (Dex) or with 3% glycerol (Glyc) in the presence or absence of varying concentrations of trichothecenes for 18 h. OD_600_ of cells treated with increasing concentrations of trichothecenes were compared to that of the untreated cells (control) to determine relative growth. The red lines indicate growth in dextrose-containing media and the blue lines indicate growth in glycerol-containing media. The green lines indicate growth of rho^0^ in dextrose-containing media. Error bars indicate S.E. where *n* = 3 independent replicates.

To determine if mitochondria were critical for sensitivity to different trichothecenes, yeast were grown in media containing a non-fermentable sugar, glycerol, which requires functional mitochondria for growth. As shown in Figure 1, the sensitivity of wild type yeast (rho+) to trichothecenes increased when cells were grown in media containing glycerol. The rho^0^ strains, devoid of functional mitochondria can only grow in media containing a fermentable sugar, such as dextrose. A rho^0^ strain derived from the wild type strain by ethidium bromide treatment was less sensitive to each trichothecene tested (Figure 1). As shown in Table 1, a decrease in the IC_50_ values for each trichothecene was observed when rho+ cells were grown on media containing glycerol compared to media containing dextrose, indicating that wild type yeast were more sensitive to trichothecenes when functional mitochondria were required for survival. Conversely, rho^0^ cells showed tolerance to significantly higher concentrations of trichothecenes, as indicated by the higher IC_50_ values (Table 1). Similar shifts in sensitivity were also observed in yeast cells treated with DON. However, yeast cells were more tolerant to DON (data not shown). These results were similar to our previous findings with the type B trichothecene, Tcin [20]. Among the trichothecenes, T-2 was more toxic than DAS, while DON had the lowest toxicity (data not shown). The type A trichothecenes are also more toxic than the type B trichothecenes to mammalian cells [26], validating the role of yeast as a relevant model to study trichothecene mechanism of action. The increased sensitivity of actively respiring yeast cells to trichothecenes and their higher tolerance when devoid of functional mitochondria suggest a critical role for mitochondria in sensitivity to type A and type B trichothecenes.

**Table 1:** IC_50_ values for trichothecenes in media containing dextrose (Dex) or glycerol (Glyc). The IC_50_ for each trichothecene was calculated using the growth curves generated from Figure 1.

	rho^+^/Dex ^1^	rho^+^/Glyc ^1^	rho^0^/Dex ^1^
T-2	95 µM	37 µM	367 µM
DAS	300 µM	139 µM	2400 µM
Tcin ^1^	2.5 µM	0.75 µM	17 µM

^1^ IC_50_ values for Tcin were based on our earlier study [20].

### 3.2. Trichothecenes Have a Direct Effect on Mitochondrial Translation

**Figure 2:**
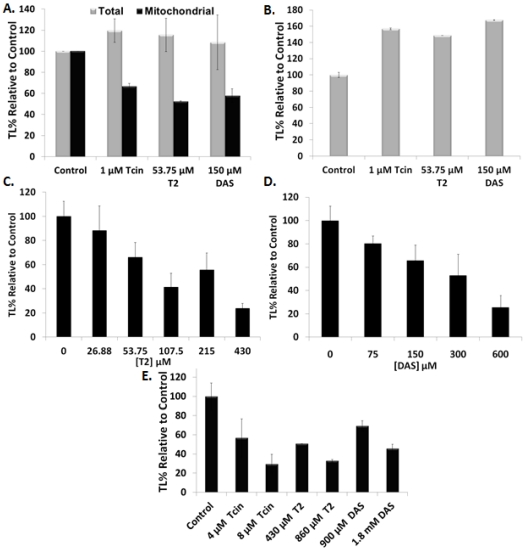
Effects of trichothecenes on total and mitochondrial protein synthesis. (**A**) Total and mitochondrial translation in wild type yeast cells treated with low doses of trichothecenes for 6 h prior to measuring [35S]-Met incorporation; (**B**) Total translation in wild type yeast cells treated with low doses of trichothecenes for 18 h prior to measuring [35S]-Met incorporation; (**C**) Mitochondrial translation in yeast cells treated with increasing concentrations of T-2 for 6 h prior to measuring [35S]-Met incorporation; (**D**) Mitochondrial translation in yeast cells treated with increasing concentrations of DAS for 6 h prior to measuring [35S]-Met incorporation; (**E**) *In organello* translation using equal amounts of mitochondria, isolated from wild type W303 yeast. ^35^[S]-methionine incorporation was measured after 10 min treatment with different concentrations of trichothecenes. Final counts (CPM) for all experiments were normalized to the OD_600_ of each sample. Translation levels of trichothecene-treated samples were expressed as a percentage of the control samples set to 100%. Error bars indicate S.E. where *n* = 3 independent replicates.

We previously showed that Tcin (type B trichothecene) inhibited mitochondrial translation to a greater extent in wild type yeast than in strains selected for trichothecene resistance, implicating mitochondrial translation as a site of action [20]. We investigated whether trichothecenes have dose-dependent effects on mitochondrial translation by varying trichothecene concentrations and the treatment period. At 6 h post treatment with low concentrations of trichothecenes total translation was largely unaffected compared to the untreated control (Figure 2A). In contrast, mitochondrial translation was inhibited 34% by 1 µM Tcin, 48% by 53.75 µM T-2, and 42% by 150 µM DAS (Figure 2A). Total translation remained uninhibited even when the treatment time was increased to 18 h (Figure 2B). A dose-dependent inhibition of mitochondrial translation was observed in intact yeast cells at 6 h post-treatment with increasing concentrations of T-2 (Figure 2C) and DAS (Figure 2D). Total translation was inhibited upon increasing the trichothecene concentrations. A 44%, 33% and 91% inhibition of total translation was observed with 215 µM T-2, 300 µM DAS and 4 µM Tcin [20], respectively at 6 h after treatment (data not shown). 

**Figure 3:**
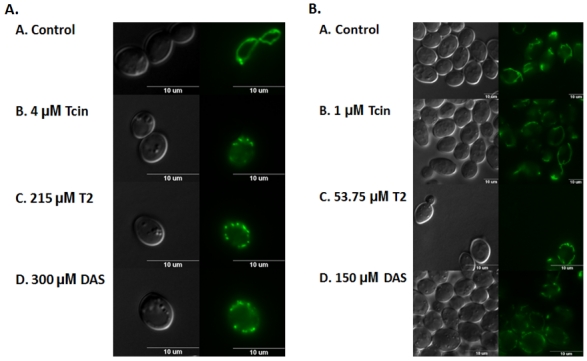
Effects of trichothecenes on mitochondrial morphology. Wild type yeast transformed with pVT100U-mtGFP, containing GFP targeted to the mitochondrial matrix was treated for 6 h with high (**A**) or low (**B**) doses of trichothecenes. Representative images are shown at 100X magnification using epifluorescence microscopy. Differential Interference Contrast (DIC) images of each cell are presented on the left (Scale bar, 10 µm).

To determine if trichothecenes have a direct effect on mitochondrial translation, we used an *in organello* assay, in which mitochondria isolated from wild type yeast (W303) [23] were treated with trichothecenes and translation was examined by [35S]-methionine incorporation [27]. As shown in Figure 2E, mitochondrial protein synthesis decreased upon increasing the concentration of trichothecenes. We observed 43% and 70% inhibition of mitochondrial translation with 4 µM and 8 µM Tcin, 49% and 67% inhibition with 430 µM and 860 µM T-2, and 30% and 55% inhibition with 900 µM and 1.8 mM DAS, respectively. The decreased sensitivity of *in organello* translation relative to mitochondrial translation in intact cells is likely due to the short duration (10 min) of treatment of the isolated mitochondria relative to the longer (6 h) treatment of intact cells. These results demonstrate that trichothecenes directly inhibit mitochondrial translation and provide evidence that inhibition of mitochondrial translation is not a secondary effect of the inhibition of cytosolic translation. 

### 3.3. Trichothecenes Cause Dose-Dependent Alteration of Mitochondrial Membrane Morphology

We previously showed that Tcin treatment led to the fragmentation of the tubular mitochondrial network in yeast, and Tcin-mediated cell death was partially rescued by mutants that regulate mitochondrial fusion and maintenance of the tubular network of mitochondria [20]. To determine if type A trichothecenes affect mitochondrial membrane morphology, we transformed wild type yeast with pVT100U-mtGFP, encoding a constitutively expressed GFP targeted to the mitochondrial matrix [25]. As shown in Figure 3A, wild type yeast showed characteristic morphology of uniformly tubular network (Figure 3A). In contrast, yeast treated for 6 h with 4 µM Tcin, 215 µM T-2, and 300 µM DAS, exhibited fragmented network of mitochondria (Figure 3A). When cells were treated with lower doses of trichothecenes (1 µM Tcin, 53.75 µM T-2, and 150 µM DAS), the mitochondrial network remained largely unaffected at 6 h after treatment (Figure 3B). However, when cells were treated for 18 h with the lower doses of trichothecenes, mitochondrial membranes showed fragmented morphology (data not shown). Although obvious changes to the mitochondrial morphology were not observed after treatment with the low doses at 6 h, mitochondrial translation was inhibited (Figure 2A). These results suggest that the inhibition of mitochondrial translation is not due to disruption of the mitochondrial membrane morphology. 

### 3.4. Trichothecenes Disrupt Mitochondrial Membrane Potential and Generation of ROS in a Dose and Time-Dependent Manner

**Figure 4:**
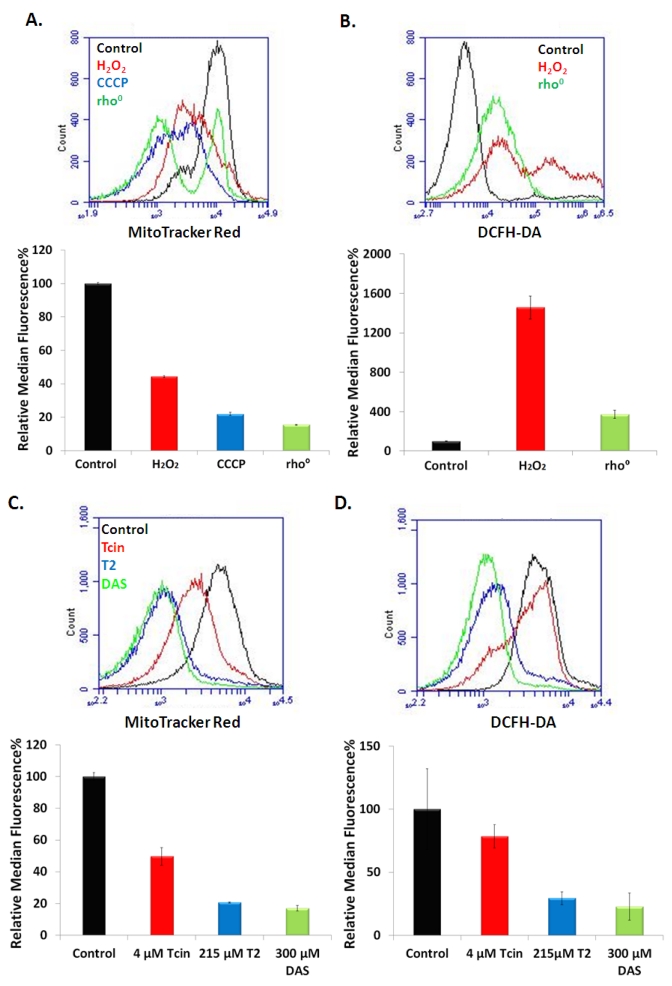
Mitochondrial membrane potential and ROS production in cells treated with trichothecenes for 6 h. Petite (rho^0^) cells and wild type (rho^+^) cells treated with 3 mM H_2_O_2_, 50 µM CCCP and stained with MitoTracker Red for changes in mitochondrial membrane potential (**A**) and DCFH-DA for ROS generation (**B**) Wild type (rho^+^) cells treated with high doses of trichothecenes and stained with MitoTracker Red for changes in mitochondrial membrane potential (**C**) and DCFH-DA for ROS generation (**D**). Median fluorescence unit for each treatment was normalized to that of the untreated control and represented as relative fluorescent unit. 25-50,000 cells from each sample were analyzed using an Accuri C6 flow cytometer. Error bars indicate S.E. where *n* = 3 independent replicates.

Deletion of genes involved in mitochondrial fusion decreased sensitivity of yeast to Tcin [20]. Inhibition of mitochondrial fusion leads to fragmentation of the mitochondria [28] and fusion defective cells show loss of mitochondrial membrane potential (ΔΨ_M_) [29]. To determine if type A and type B trichothecenes affect ΔΨ_M_, we analyzed yeast cells stained with MitoTracker Red using flow cytometry. Wild type cells stained with MitoTracker Red exhibited an active mitochondrial membrane potential observed as an increase in MitoTracker Red staining (Figure 4A). In contrast, in H_2_O_2_-treated cells, ΔΨ_M_ decreased to 44% relative to the control (Figure 4A). Treatment of the wild type cells with the decoupling agent, carbonyl cyanide 3-chlorophenylhydrazone (CCCP) decreased the ΔΨ_M_ to 22% due to depolarization of the mitochondria [30] (Figure 4A). As expected, the rho^0^ cells showed only 15% staining with MitoTracker Red (Figure 4A) due to the absence of functional mitochondria. A 50%, 79%, and 83% drop in ΔΨ_M_ was observed after treatment with 4 µM Tcin, 215 µM T-2, and 300 µM DAS, respectively for 6 h (Figure 4C). This drop in ΔΨ_M_ corresponds to the mitochondrial membrane fragmentation observed at six hours after treatment with the same doses of trichothecenes, indicating that the loss of ΔΨ_M_ correlates with the fragmented morphology of the mitochondria (Figure 3A). 

Wild type cells stained for ROS production using 2',7'-dichlorfluorescein-diacetate (DCFH-DA) [31] showed very low levels of ROS (Figure 4B). In contrast, H_2_O_2_-treated cells exhibited a significant increase in ROS production relative to the control, as indicated by an increase in DCFH-DA staining (Figure 4B). ROS levels decreased by 21%, 70%, and 77% in cells treated with 4 µM Tcin, 215 µM T-2, and 300 µM DAS, respectively (Figure 4D), in agreement with the fragmented mitochondrial morphology (Figure 3A) and the reduced ΔΨ_M_ (Figure 4A). These results indicate that at high doses, trichothecenes promote depolarization of the mitochondrial membranes and inhibit ROS production.

**Figure 5:**
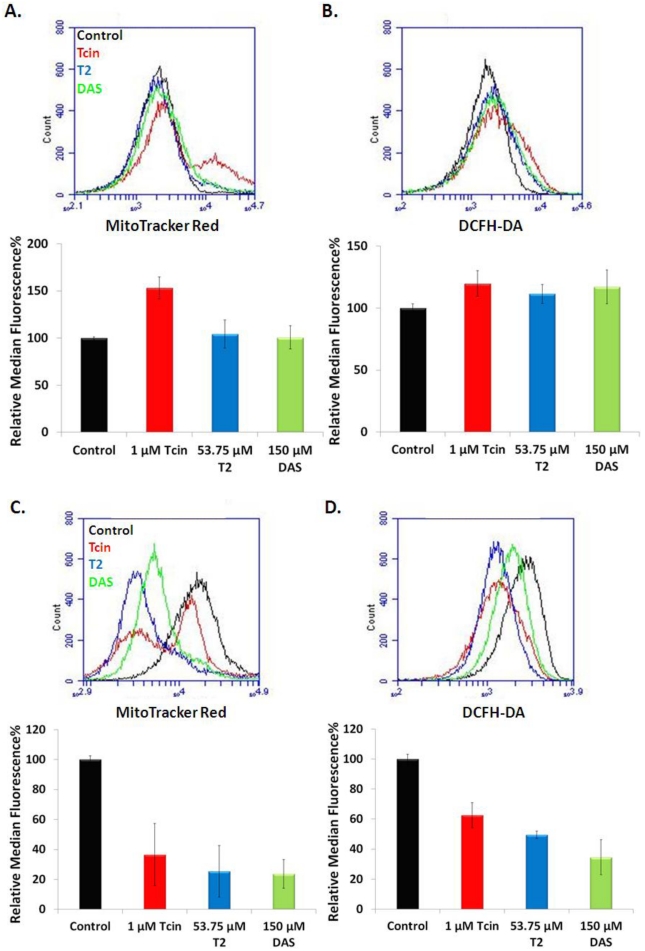
Mitochondrial membrane potential and ROS production and in cells treated with trichothecenes for 6 h and 18 h at low doses, which inhibit mitochondrial, but not total translation. Wild type yeast, treated with trichothecenes for 6 h and stained with MitoTracker Red for changes in mitochondrial membrane potential (**A**) and DCFH-DA for ROS generation (**B**). At 18 h post treatment with trichothecenes, wild type yeast cells were stained with MitoTracker Red for changes in mitochondrial membrane potential (**C**) and DCFH-DA for ROS generation (**D**). Median fluorescence unit for each treatment was normalized to that of the untreated control and represented as relative fluorescent unit. 25-50,000 cells from each sample were analyzed using an Accuri C6 flow cytometer. Error bars indicate S.E. where *n* = 3 independent replicates.

The effects of trichothecenes on ROS production and ΔΨ_M_ were time-dependent when cells were treated with low doses of trichothecenes. At 6 h post-treatment a 53% increase in ΔΨ_M_ was observed after treatment with 1 µM Tcin, but not with 53.75 µM T-2 or 150 µM DAS (Figure 5A). A 20%, 12%, 18% increase in ROS production was observed after treatment with 1 µM Tcin, 53.75 µM T-2 and 150 µM DAS for 6 h, respectively (Figure 5B). However, when the duration of the treatment was increased from 6 to 18 h, a decrease in ΔΨ_M_ (Figure 5C) and ROS levels (Figure 5D) was observed. At 18 h post treatment, a 63%, 75%, and 76% drop in ΔΨ_M_ and a 37%, 50%, and 65% drop in ROS generation was observed in cells treated with 1 µM Tcin, 53.75 µM T-2, and 150 µM DAS, respectively. This was confirmed by fluorescence microscopy, which showed fragmentation of the mitochondrial membrane as well as a decrease in the uptake of the MitoTracker Red in yeast treated with the different trichothecenes for 18 h (data not shown). These results indicate that the effects of trichothecenes on ΔΨ_M_ and ROS generation are dose and time-dependent. Although mitochondrial membrane integrity was not compromised at 6 h post treatment with the low doses of trichothecenes, mitochondrial translation was significantly inhibited, suggesting that the inhibition of mitochondrial translation is not a consequence of the damage to the mitochondrial membrane integrity. Moreover, ΔΨ_M_ and ROS production were inhibited and mitochondrial morphology was altered after treatment of yeast with the low doses of trichothecenes for 18 h (Figure 5C,D) when total translation was not inhibited (Figure 2B), suggesting that the effect of trichothecenes on mitochondrial membrane integrity is not a secondary effect of the inhibition of total translation. 

## 4. Discussion

### 4.1. Inhibition of Mitochondrial Translation by Trichothecenes Is Not a Secondary Effect of the Inhibition of Cytosolic Translation

Trichothecenes induce many cellular and physiological changes and it is likely that they have multiple cellular targets. However, the mechanism of trichothecene toxicity is not well understood. In this study, we examined the effect of type A (T-2 and DAS) and B (Tcin) trichothecenes on mitochondrial translation and membrane integrity. Wild type yeast treated with trichothecenes showed an increase in growth sensitivity when functional mitochondria were required for growth (Figure 1). Yeast cells without functional mitochondria remained largely resistant to otherwise lethal doses of trichothecenes (Figure 1). Taken together these results provide evidence that mitochondria play a critical role in the sensitivity to type A and type B trichothecenes and suggest that trichothecenes may inhibit a mitochondrial function essential for growth that depends on respiratory activity.

To determine if trichothecenes affect mitochondrial translation, we examined inhibition of total and mitochondrial translation after exposure of yeast cells to increasing doses of trichothecenes. When yeast cells were treated with low doses of trichothecenes, total translation was not inhibited. However, mitochondrial translation was inhibited (Figure 2A). Dose-dependent inhibition of mitochondrial translation was observed with increasing concentrations of T-2 (Figure 2C) and DAS (Figure 2D), suggesting that inhibition of mitochondrial translation was not a secondary effect of the inhibition of total translation. To determine if trichothecenes have a direct effect on mitochondrial translation, we isolated yeast mitochondria and examined *in organello* translation. Translation was inhibited in isolated yeast mitochondria after treatment with increasing concentrations of type A and type B trichothecenes (Figure 2E). These results indicate that trichothecenes have a dose-dependent effect on mitochondrial protein synthesis independent of their effects on cytosolic translation.

### 4.2. Inhibition of Mitochondrial Translation by Trichothecenes Is Not Due to Mitochondrial Membrane Damage

The inhibition of mitochondrial translation could be due to the damage to the mitochondrial membranes, which might render mitochondria nonfunctional. To address this possibility, we investigated the effects of type A and B trichothecenes on mitochondrial morphology. The tubular mitochondrial network, characteristic of actively respiring cells, was severely fragmented 6 h after treatment of yeast with the high doses of trichothecenes (Figure 3A), but not after treatment with the low doses (Figure 3B), which were inhibitory to mitochondrial translation. These results suggested that inhibition of mitochondrial translation was not due to the effects of trichothecenes on the mitochondrial membrane morphology. 

To further investigate the effects of trichothecenes on mitochondrial membranes, we measured ΔΨ_M_ and ROS levels, two biomarkers for mitochondrial integrity. T-2 toxin was previously reported to promote severe damage to the DNA via generation of ROS in both yeast and mammals [18,19,32]. Mitochondrial ROS generation is believed to be dependent on mitochondrial membrane potential. Studies involving cell death induced by chemicals such as hydrogen peroxide and acetic acid in yeast [33,34,35,36] showed that prior to ROS generation the mitochondrial membrane becomes hyperpolarized leading to excess ROS production followed by depolarization of the mitochondrial membrane [37,38]. A depolarized mitochondrial membrane can also lead to the fragmentation of the mitochondrial tubular network [39,40,41]. 

Unlike actively respiring cells, which exhibited an active ΔΨ_M_ (Figure 4A) and low ROS production (Figure 4B), cells treated with high doses of trichothecenes showed a decrease in ΔΨ_M_ (Figure 4C). A corresponding decrease in ROS levels was also observed in yeast treated with the high doses of trichothecenes (Figure 4D). The fragmented mitochondrial network, which is observed after treatment with the high doses of trichothecenes (Figure 3A) could therefore be due to the loss of the mitochondrial membrane potential. 

Mitochondrial translation was inhibited when yeast cells were treated with low doses of trichothecenes for 6 h (Figure 2A) when the cells maintained the tubular mitochondrial morphology (Figure 3B), an active ΔΨ_M_ (Figure 5A). These results suggest that mitochondrial translation inhibition is independent of damage to the mitochondrial membranes. 

### 4.3. Mitochondrial Membrane Damage by Trichothecenes Is Not a Secondary Effect of the Inhibition of Total Translation

To determine if mitochondrial membranes were affected due to inhibition of cytosolic translation, we examined ΔΨ_M_ and ROS levels in cells treated with the low doses of trichothecenes, which did not inhibit total translation. ΔΨ_M_ increased in cells treated with low doses of Tcin at 6 h after treatment (Figure 5A). Moderate increases in ROS levels (Figure 5B) were also observed in yeast treated with Tcin, T-2 and DAS for 6 h. However, when the duration of the treatment was increased from 6 to 18 h, ΔΨ_M_ (Figure 5C) and ROS levels (Figure 5D) decreased corresponding to a fragmented mitochondrial network (data not shown). The decrease in ΔΨ_M_ observed at 18 h could be due to an increase in ROS that occurred earlier. ROS levels may begin to drop as the mitochondrial membrane becomes depolarized by the trichothecenes. In a previous study, Chaudhari *et al.* [xref^32^] observed an increase in ROS levels in human cervical cancer cells within 30 min of treatment with T-2, followed by a decrease in ROS levels at 4 h after treatment [32]. The fragmented mitochondrial network, which is observed after treatment with the low doses of trichothecenes for 18 h could be due to the loss of the mitochondrial membrane potential. Since total translation was not inhibited at 18 h after treatment with the low doses (Figure 2B), the effect of trichothecenes on mitochondrial membrane integrity was not secondary to the inhibition of total translation. The mitochondrial membrane damage may occur downstream of the inhibition of mitochondrial translation. Alternatively, trichothecenes may have a direct effect on mitochondrial membranes, leading to the loss of mitochondrial membrane potential.

Recent studies have implicated mitochondria as a target of other toxins. A type I RIP, saporin-6 was shown to specifically cleave the human mitochondrial DNA D-loop [42]. Mitochondrial dysfunction was implicated in the toxicity of other *Fusarium* mycotoxins, such as enniatins [43], and fumonisin B1 [44]. Our results indicate that mitochondrial translation is a primary target of trichothecenes and is not inhibited secondary to membrane damage. In light of our findings, it will be of significant importance to include the mitochondrial translation machinery as a potential target for engineering crop plants resistant to trichothecenes. It will also be important to investigate the effects of trichothecenes on mitochondria from higher eukaryotes and determine how the pathogen itself protects its own mitochondria from the deleterious effects of these toxins. 

## 5. Conclusions

The present work shows that type A and type B trichothecenes inhibit mitochondrial translation independent of their effects on cytosolic translation and mitochondrial membrane integrity. Inhibition of mitochondrial translation represents a novel mode of action for trichothecene mycotoxins and a potential target for developing protection strategies.
